# Mitonuclear interactions modulate nutritional preference

**DOI:** 10.1098/rsbl.2023.0375

**Published:** 2023-12-06

**Authors:** M. Florencia Camus, Sahutchai Inwongwan

**Affiliations:** ^1^ Research Department of Genetics, Evolution and Environment, University College London, Gower Street, London, WC1E 6BT, UK; ^2^ Department of Biology, Faculty of Science, Chiang Mai University, Chiang Mai, Thailand; ^3^ Research Center of Deep Technology in Beekeeping and Bee Products for Sustainable Development Goals, Chiang Mai University, Chiang Mai, Thailand

**Keywords:** mtDNA, mitonuclear epistasis, nutrition, behaviour

## Abstract

In nature, organisms are faced with constant nutritional options which fuel key life-history traits. Studies have shown that species can actively make nutritional decisions based on internal and external cues. Metabolism itself is underpinned by complex genomic interactions involving components from both nuclear and mitochondrial genomes. Products from these two genomes must coordinate how nutrients are extracted, used and recycled. Given the complicated nature of metabolism, it is not well understood how nutritional choices are affected by mitonuclear interactions. This is under the rationale that changes in genomic interactions will affect metabolic flux and change physiological requirements. To this end we used a large *Drosophila* mitonuclear genetic panel, comprising nine isogenic nuclear genomes coupled to nine mitochondrial haplotypes, giving a total of 81 different mitonuclear genotypes. We use a capillary-based feeding assay to screen this panel for dietary preference between carbohydrate and protein. We find significant mitonuclear interactions modulating nutritional choices, with these epistatic interactions also being dependent on sex. Our findings support the notion that complex genomic interactions can place a constraint on metabolic flux. This work gives us deeper insights into how key metabolic interactions can have broad implications on behaviour.

## 1. Introduction

To thrive, organisms exploit resources offered by the environment. Distinct niches will contain different nutritional resources, with these resources further varying in space and time. While the environment may unexpectedly change, organisms make constant decisions regarding what nutrients are required to maximize physiological and reproductive function. Previous work has demonstrated that nutritional choices depend largely on internal physiology, with a classic example being behavioural changes following mating in insect species [1,2].

For instance in *Drosophila,* female egg production (reproductive output) has been directly linked to the nutritional state of female flies, with flies having a greater reproductive output on diets containing higher protein concentrations [3]. This change in reproduction creates a large shift in internal physiology, whereby metabolism is being re-wired to incorporate the new role of producing high-quality eggs (which are rich in protein). Consequently, to meet these new physiological requirements, females alter their nutritional decision-making, and chose to eat diets richer in protein [2]. These results allude to both a metabolic and behavioural shift for diet preference in response to a change in physiological state [1,4].

Mitochondria are the hub of both energy transduction and intermediary metabolism (sum of all intracellular chemical processes that turn nutrition into energy) [5]. Perturbations in cell respiration have been shown to have downstream implications for biosynthesis, signalling, gene expression and life-history phenotypes [6]. Furthermore, proteins involved in respiratory complexes (OXPHOS) are encoded by two genomes: mitochondrial (mtDNA) and nuclear [7]. This means that for efficient organismal respiration, both genomes need to work harmoniously for proper assembly and function of the electron transport chain. Despite their central importance, mitochondria are uniquely vulnerable to disruption, as both genomes have very different rates and modes of evolution [8–10]. While the nuclear genome is diploid, sexual and large, mtDNA genomes are small, circular and with strict uniparental inheritance across most species [11]. Serious incompatibilities between genomes have been found in natural populations of several species, generated through introgression between divergent populations [12–16]. We can therefore predict that mitonuclear epistasis has the capability to constrain metabolic phenotypes.

It is expected that alterations to mitochondrial function are a critical factor influencing internal physiology. Indeed, recent work has demonstrated that *Drosophila* with a specific Complex I mtDNA mutation rewire physiology, diverting energy production via beta-oxidation and partially avoiding Complex I [17]. Nutritional requirements have also been found to be dependent on the mtDNA genotype [18], indicating that these inter-genomic interactions can modulate physiology and life-history. Here we examine how internal physiology, mediated by mitonuclear interactions, can alter diet choice. We use a previously created panel of 81 genotypes, which differ in their inter-genomic combinations [19]. Our results demonstrate that there are complex epistatic interactions dictating nutritional choice.

## 2. Material and methods

### (a) *Drosophila* stock and maintenance and mitonuclear panel

The mitonuclear *Drosophila* panel aims to capture a range of genetic diversity analogous to that observed among fruit fly populations globally. It was generated by a full factorial design using nine sets of isogenic lines, combining nuclear DNA (nuDNA) with mtDNA from each line [19–21]. Nuclear genomes were replaced using a known balancer chromosome crossing scheme [10]. The sources for these genetic lines are as follows: **A**/ZIM184 (Zimbabwe), **B**/B04 (Beijing), **C**/I16 (Ithaca), **D**/I23 (Ithaca), **E**/N14 (Netherlands), **F**/N15 (Netherlands), **G**/T01 (Tasmania), **H**/T23 (Tasmania), **i**/N01 (Netherlands). Since the creation of the panel, each line has been further backcrossed to its desired nuclear genome every four generations for the past 2 years. Notably, due to a pronounced incompatibility, one specific mitonuclear genotype (F_nuc_ × A_mito_) could not be sustained. Consequently, all subsequent experiments were conducted using a subset of 80 viable genotypes.

Lines are propagated by 4-day-old parental flies, with approximate densities of 80–100 eggs per vial. Flies are reared on 8 ml of sugar–yeast agar medium per vial (see electronic supplementary material, table S1 for recipe), with ad libitum live yeast added to each vial to promote female fecundity. Furthermore all stocks are kept at 25°C and 50% humidity, on a 12 : 12 h light : dark cycle. All lines have been cleared of potential bacterial endosymbionts, such as *Wolbachia*, through a tetracycline treatment at the time the lines were created. Clearance was verified using *Wolbachia*-specific PCR primers [22].

### (b) Synthetic diet

We used a modified liquid version of a holidic diet [23], prepared entirely from synthetic components (for recipes see electronic supplementary material, tables S2–S4). We created two diet stocks, each containing either a protein or carbohydrate source, with each stock also having all nutritional components (i.e. vitamins, minerals, lipids) at equal concentration. The protein stock composition consisted of a mixture of all amino acids, plus a 20% suspension of dried yeast extract, made at the same protein concentration as the synthetic solution (electronic supplementary material, table S2–S4), whereas the carbohydrate stock contained sucrose. We added yeast extract to our protein stock, because our preliminary data show that synthetic amino acids are not enticing to the flies and they chose not to eat it [2]. Given that yeast extract also contains sugars, the final protein diet then included 4% carbohydrate. It is noteworthy to mention that most diet preference experiments use solely yeast as a protein source [24], and although our protein stock is mostly synthetic, it is a step in the right direction. These two diets were presented to the flies during the experimental trial in separate capillary tubes.

### (c) Dietary preference assay

Flies from each genotype were collected 24 h following eclosion and kept in SY vials for 48 h. This period with the opposite sex made sure that all flies had mated. Following this period, flies were split by sex and three same-sex flies were transferred to new vials containing an 0.8% agar–water mixture. We set up five vials of triplets per sex and genotype combination. Agar–water vials provide water for the flies, but have no nutritional value. Furthermore, we use triplets of flies to minimize between-vial variance [25]. Flies were kept in a controlled temperature room (25°C), 12 L : 12 D light cycle and high relative humidity greater than 80%.

In accordance with previous literature using this methodology [25,26], flies were kept in agar–water vials overnight, then supplied with two 5 µl microcapillary tubes (ringcaps, Hirschmann); one containing the protein solution and the other the carbohydrate solution. Every day, capillary tubes were substituted for new ones, and the amount of food consumed by each group of three flies was documented over a span of 3 days. As a comparative benchmark, the pace at which the diet solutions evaporated was gauged by using vials that contained the capillary tubes filled with the solutions, akin to the experimental set-up, but no flies were present. These vials were positioned randomly in the temperature-regulated room. The mean evaporation per day from these control vials was employed to adjust the recorded diet consumption, accounting for the effects of evaporation.

### (d) Statistical analyses

To determine if dietary choices depend on sex and mitonuclear genotype, we used a multivariate analysis of variance (MANOVA) to analyse nutritional preference data. In our MANOVA, the main model had protein and carbohydrate as response variables, with all possible interactions between, sex, nuclear and mitochondrial genotype as fixed effects. All analyses were performed in R v. 3.3.2 [27]. With this analysis, we can quantify the joint response of nutrition, but also look at protein and carbohydrate via a univariate analysis.

We also chose to use a slightly different analysis method [2], where we calculate the quantity and quality of food consumed (electronic supplementary material, figure S1). This method uses trigonometry to calculate:

(1) the length of a vector from the origin of the plot to a given datapoint. This is a measure of total food consumed by the fly triplet.
(2) the inner angle created by this vector. This is a measure of quality of food consumed, with a smaller angle (*α* < 45°) indicating that flies prefer to eat a more protein-rich diet, and a larger angle (*α* > 45°) indicating that flies prefer higher carbohydrate-rich diets.

After calculating these values for all our genotypes, we used general linear models, with angle or length of vector as a response variable with all possible interactions between, sex, nuclear and mitochondrial genotype as fixed effects.

## 3. Results

**Figure 1.**
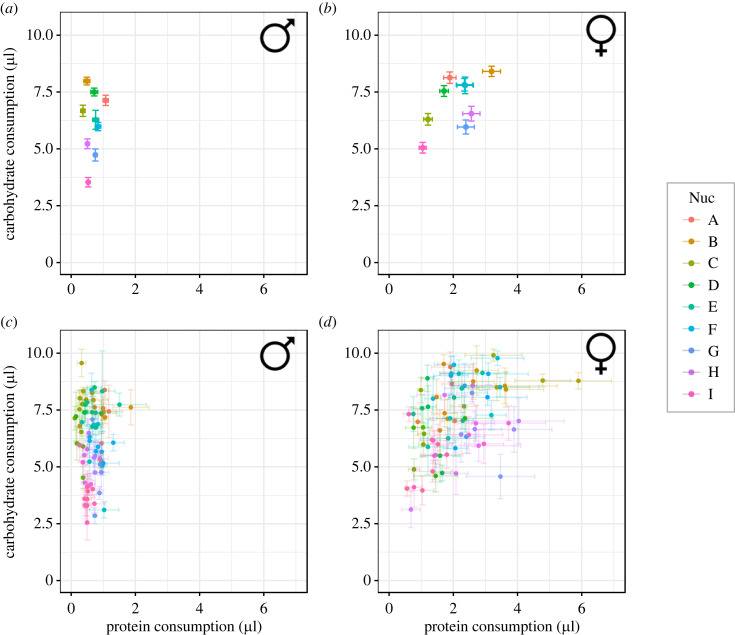
Nutritional preferences across mitonuclear genotypes. Genotype-specific dietary response to mating for females (right) and males (left), measured as the intake (mean ± SE) of protein (*x*-axis) and carbohydrate (*y*-axis). Panel (*a*) condenses all mtDNA genotypes, grouping them by nuclear genetic variation, whereas panel (*b*) shows all datapoints, colouring them by nuclear background.

We find diet choice to be an overall sexually dimorphic trait, with females consuming more protein and carbohydrate than males (Pillai's trace = 0.32582, approx. *F* = 169.151, *p* < 0.0001, tables 1 and 2, figure 1). Nuclear genotype had an important role in modulating nutritional choices (Pillai's trace = 0.48788, approx. *F* = 28.272, *p* < 0.0001, figure 1*a*), but these were contingent on sex (sex × nuDNA: Pillai's trace = 0.14819, approx. *F* = 7.012, *p* < 0.0001). When considering the nuclear genotype, all females consumed more protein than males, but increased consumption of carbohydrate (compared to males) was dependent on the genetic background.

**Table 1.** Results from (*a*) multivariate (MANOVA) and (*b*) univariate (ANOVA) outputs for each sex. Response variables are mitonuclear genotype.

(*a*) multivariate						
	d.f.	Pillai	approx. F	numDF	denDF	Pr(>F)
sex	1	0.32582	169.151	2	700	< 0.001
mtDNA	8	0.02895	1.287	16	1402	0.1967
nuDNA	8	0.48788	28.272	16	1402	< 0.001
sex × mtDNA	8	0.03103	1.381	16	1402	0.1421
sex × nuDNA	8	0.14819	7.012	16	1402	< 0.001
mtDNA × nuDNA	62	0.33536	2.278	124	1402	< 0.001
sex × mtDNA × nuDNA	62	0.26621	1.736	124	1402	< 0.001
residuals	701					
(*b*) univariate						
	d.f.	Sum S	Mean Sq	F	Pr(<F)	
protein						
sex	1	418.09	418.09	306.3792	< 0.001	
mtDNA	8	11.37	1.42	1.0417	0.4028	
nuDNA	8	127.02	15.88	11.6346	< 0.001	
sex × mtDNA	8	8.79	1.1	0.805	0.5983	
sex × nuDNA	8	96.08	12.01	8.8011	< 0.001	
mtDNA × nuDNA	62	139.09	2.24	1.644	0.0019	
sex × mtDNA × nuDNA	62	107.27	1.73	1.2679	0.0869	
residuals	701	956.6	1.36			
carbohydrate						
sex	1	180.19	180.193	71.3874	< 0.001	
mtDNA	8	32.14	4.018	1.5919	0.1235	
nuDNA	8	1180.5	147.563	58.4604	< 0.001	
sex × mtDNA	8	37.52	4.69	1.8581	0.0637	
sex × nuDNA	8	102.29	12.786	5.0656	< 0.001	
mtDNA × nuDNA	62	501.91	8.095	3.2072	< 0.001	
sex × mtDNA × nuDNA	62	340.76	5.496	2.1774	< 0.001	
residuals	701	1769.43	2.524

**Table 2.** Linear model results from trigonometric analysis of quantity (length) and quality (angle) of food consumed.

measurement	d.f.	sum Sq	mean Sq	F	Pr(>F)
length of vector					
sex	1	359.28	359.28	130.8332	< 0.001
mtDNA	8	1252.22	156.53	56.9996	< 0.001
nuDNA	8	48.14	6.02	2.1913	0.026
sex × mtDNA	8	124.48	15.56	5.6662	< 0.001
sex × nuDNA	8	36.4	4.55	1.6569	0.106
mtDNA × nuDNA	62	551.16	8.89	3.2372	< 0.001
sex × mtDNA × nuDNA	62	340.81	5.5	2.0017	< 0.001
residuals	701	1925.03	2.75		
angle of vector					
sex	1	16776	16776.2	231.0244	< 0.001
mtDNA	8	4336	542	7.4637	< 0.001
nuDNA	8	278	34.8	0.4793	0.8713
sex × mtDNA	8	4294	536.8	7.3924	< 0.001
sex × nuDNA	8	681	85.2	1.173	0.3127
mtDNA × nuDNA	62	6189	99.8	1.3747	0.0339
sex × mtDNA × nuDNA	62	5627	90.8	1.2498	0.1005
residuals	701	50904	72.6		

The effect of mitochondrial genotype was also an important contributor to diet choices across our strains. While there was no main significant effect for mtDNA, we found significant mitonuclear effects (Pillai's trace = 0.33536, approx. *F* = 2.278, *p* < 0.0001, table 1) and a three-way interaction effect with sex (Pillai's trace = 0.26621, approx. *F* = 1.736, *p* < 0.0001, table 1, figure 1*b*, electronic supplementary material, figure S2).

**Figure 2.**
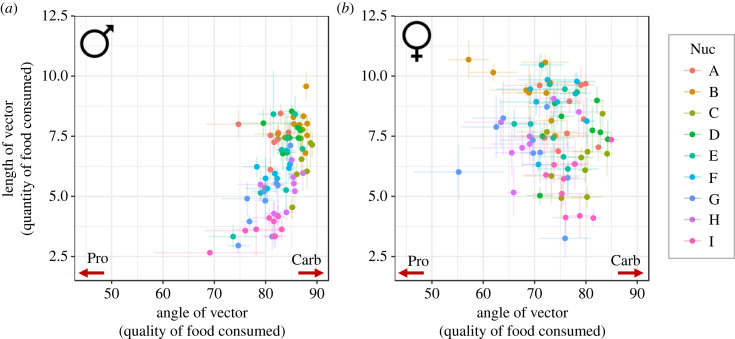
Genotype-specific dietary responses for males (left) and females (right) measured as the mean ± SE of angle (food quality, *x*-axis) and length (food quantity, *y*-axis) of the genotype-specific vector.

We analysed this dataset using a trigonometric approach to get better insights into diet quantity (length of vector) and quality (angle of vector). Our analyses show a significant three-way interaction between mtDNA, nuDNA and sex for quantity of food consumed (*F* = 2.0017, *p* < 0.001, figure 2). We did not find such complex interactions for diet quality, with an overall sex effect (*F* = 231.024, *p* < 0.001, figure 2), nuclear effect (*F* = 2.1913, *p* = 0.026, figure 2), and an interaction between sex and nuclear genotype (*F* = 7.392, *p* < 0.001, figure 2). Furthermore, we found an interaction between mtDNA and nuDNA (*F* = 1.375, *p* = 0.339, figure 2).

We further examined cross-sex genetic correlations for protein and carbohydrate consumption (electronic supplementary material, figure S3). While we find no significant genetic correlation between males and females for protein consumption (*r* = 0.081, *p* = 0.475), we see a significant positive correlation for carbohydrate consumption (*r* = 0.518, *p* < 0.001).

## 4. Discussion

We explored changes in dietary preference across a panel of *Drosophila* lines that differed in their mitonuclear genotype. Our results confirm previously obtained results whereby diet choice is a sexually dimorphic trait, with females eating more than males [28,29]. This is hypothesized to be due to the differences in reproductive strategies and outputs, with female fitness largely comprised of the quality/quantity of oocytes produced, which is thought to require more protein [30]. Notably we find nutritional choices not only influenced by sex, but significantly affected by mitonuclear genotypes. For instance, some mitonuclear combinations decrease the sexual dimorphism to the point where males and females have similar phenotypes. These data suggest all these complex intergenomic interactions impact metabolism, internal physiology and feeding behaviour. We need to note that although our protein diet was composed mostly of purified amino acids, it contained 4% carbohydrate from yeast extract. Previous work established that flies do not eat purified amino acids by themselves, hence we had to spike the protein stock with a small amount of lyophilized yeast. While we cannot fully disentangle the possible effects of carbohydrate in our protein diet, that diet has a much greater protein concentration (greater than 90%: amino acids, vitamins/minerals/lipid buffer, yeast extract) than all previous studies (approx. 42%: yeast extract) and thus our study is more informative about the effects of protein on dietary preference.

The greatest genetic effect we observed was contributed from the nuclear genome, which is expected, as the nuclear genome contributes thousands of genes, compared to the 37 genes encoded in the mtDNA [31]. Previous work has shown nuclear genetic variation for diet choice and nutritional requirements [26,32]. For instance, Reddiex and collegues used quantitative genetics techniques to understand to degree to which organisms can respond to selection using the Drosophila Genetic Resource Panel (DGRP) [26]. These results suggest that there are many evolutionary forces in action maintaining this variation. These forces include sexual conflict/antagonistic selection [26], epistatic interactions [33–35] and/or balancing selection via temporal variation in environmental conditions [36,37]. Furthermore, there have been several studies examining how mitonuclear epistasis interacts with the nutritional environment to elicit life-history responses. These studies include Zhu *et al*. [10], where authors found mitonuclear interactions impacted ageing under caloric restriction, with certain mitonuclear genotypes being more impacted than others. More recently, Dobson *et al.* [38] demonstrated that parental effects of dietary lipid and amino acid variation on offspring fitness is modulated by mitonuclear interactions. Furthermore, Aw *et al*. [17] examined how mitonuclear interactions shaped metabolic flexibility to diet, with certain genetic combinations constraining metabolism on certain nutrients. These genetic constraints resulted in metabolic flux being rewired to avoid placing a larger burden on mitochondrial metabolism and obtaining energy through other pathways [17]. Likewise, work in the medical field has shown that some symptoms of mitochondrial disorders can be ameliorated by bypassing mitochondrial metabolism via ingestion of ketogenic diets [39]. What is currently not fully studied is how these metabolic changes modulate behavioural responses.

The notion that internal physiology alters behaviour has been proposed over a decade ago [40], with the classic study system being physiological changes relating to the transition from virgin state to reproductive female [1,23,41]. Further work has shown nuclear genetic variation can also alter nutritional choices, indicating that there are both genetic and environmental contributors to this trait [26]. Our results show that interactions between mitonuclear interactions are important for modulating behavioural nutritional responses. These findings also support the notion that mitochondria are not solely energy producers, but also the serve as crucial signalling organelles [42]. While there are many pathways that are involved in mitochondrial signal transduction [43], the first logical place to investigate would be nutrient sensing pathways [44]. Further work should investigate this question on two fronts. First, how these nutritional decisions impact key life-history traits, with components of fitness being a key exemplar. And second, how these complex genomic interactions modulate changes in bioenergetic and metabolic fluxes across tissues, including investigating changes in neuronal metabolic profiles, which could explain how an internal physiological signal translates to behavioural phenotypic expression.

## Data Availability

All data are attached to the submission in the electronic supplementary material files [45]. Data are available from the Dryad Digital Repository: https://doi.org/10.5061/dryad.t4b8gtj7t [46].
